# Autophagy inhibition radiosensitizes *in vitro*, yet reduces radioresponses *in vivo* due to deficient immunogenic signalling

**DOI:** 10.1038/cdd.2013.124

**Published:** 2013-09-13

**Authors:** A Ko, A Kanehisa, I Martins, L Senovilla, C Chargari, D Dugue, G Mariño, O Kepp, M Michaud, J-L Perfettini, G Kroemer, E Deutsch

**Affiliations:** 1 INSERM U1030, Radiothérapie moléculaire SIRIC SOCRATES, LABEX LERMIT & DHU TORINO, Institut Gustave Roussy, Villejuif, France; 2 Gustave Roussy Cancer Campus, Villejuif, France; 3 SIRIC SOCRATES, LABEX LERMIT & DHU TORINO, Université Paris Sud—Paris 11, Villejuif, France; 4 INSERM U848, Institut Gustave Roussy, Villejuif, France; 5 Metabolomics and Cell Biology Platforms, Institut Gustave Roussy, Villejuif, France; 6 Equipe 11 labellisée Ligue contre le Cancer, Centre de Recherche des Cordeliers, Paris, France; 7 Pôle de Biologie, Hôpital Européen Georges Pompidou, AP-HP, Paris, France; 8 Université Paris Descartes, Paris 5, Paris, France; 9 Department of Radiation Oncology, Institut Gustave Roussy, Villejuif, France

**Keywords:** radiotherapy, irradiation, autophagy, non-small-cell lung carcinoma, immunogenic cell death

## Abstract

Clinical oncology heavily relies on the use of radiotherapy, which often leads to merely transient responses that are followed by local or distant relapse. The molecular mechanisms explaining radioresistance are largely elusive. Here, we identified a dual role of autophagy in the response of cancer cells to ionizing radiation. On one hand, we observed that the depletion of essential autophagy-relevant gene products, such as ATG5 and Beclin 1, increased the sensitivity of human or mouse cancer cell lines to irradiation, both *in vitro* (where autophagy inhibition increased radiation-induced cell death and decreased clonogenic survival) and *in vivo*, after transplantation of the cell lines into immunodeficient mice (where autophagy inhibition potentiated the tumour growth-inhibitory effect of radiotherapy). On the other hand, when tumour proficient or deficient for autophagy were implanted in immunocompetent mice, it turned out that defective autophagy reduced the efficacy of radiotherapy. Indeed, radiotherapy elicited an anti-cancer immune response that was dependent on autophagy-induced ATP release from stressed or dying tumour cells and was characterized by dense lymphocyte infiltration of the tumour bed. Intratumoural injection of an ecto-ATPase inhibitor restored the immune infiltration of autophagy-deficient tumours post radiotherapy and improved the growth-inhibitory effect of ionizing irradiation. Altogether, our results reveal that beyond its cytoprotective function, autophagy confers immunogenic properties to tumours, hence amplifying the efficacy of radiotherapy in an immunocompetent context. This has far-reaching implications for the development of pharmacological radiosensitizers.

Radiation is a widely used therapeutic approach for the loco-regional management of numerous cancerous malignancies. Depending on the location and tumour stage, radiation therapy can be administered alone or in combination with surgery, chemotherapy and/or hormonal treatments. In response to irradiation, tumour cells can die through different mechanisms such as apoptosis, necrosis and mitotic catastrophe,^1, 2^ which in many cases are preceded or accompanied by pro-survival processes such as autophagy.^3^ The type of cellular response(s) and its relative contribution to the anti-tumour effect depends on several factors. In fact, intrinsic parameters such as the tumour cellular type, the cell cycle phase in which the irradiated cells are, as well as treatment protocol parameters, such as radiation dose and treatment intervals, may strongly influence the tumour response to irradiation.^4^

Macroautophagy (herein referred to as ‘autophagy') involves the highly regulated sequestration of cytoplasmic organelles or bulk portions of cytosol inside two-membraned vesicules, named autophagosomes, which eventually fuse with lysosomes for the degradation of their inner autophagosome membrane and luminal content.^5^ Autophagy has an essential role in cellular adaptation to multiple types of stress, recycling of superfluous or damaged cellular material, quality control of organelles, removal of protein aggregates and destruction of intracellular pathogens.^6, 7^ The discovery of autophagy-related genes (ATG) in the yeast *Saccharomyces cerevisiae* (reviewed in Klionsky *et al.*^8^) has facilitated the molecular dissection of autophagy and participated to the better understanding of pathophysiological role of autophagy, including the generation, progression and therapeutic response of cancers.^9^ The formation of the autophagosome is based on subsequent protein conjugation steps.^10^ The initial formation of ATG5–ATG12 complexes allows for the recruitment of ATG8 (LC3 in mammalian cells) by phosphatidylethanolamine (PE). ATG5–ATG12 complexes are recycled to the cytosol before the complete formation of the autophagosome. ATG6 (Beclin 1 in mammalian cells) interacts with and activates phosphatidylinositol 3-phosphate kinase type III, thus generating phosphatidylinositol 3-phosphate-enriched membranes, which contribute to the recruitment of downstream autophagic factors.^11^ In tumour cells, autophagy acts as a double-edged sword. On one hand, autophagy is a haploinsufficient tumour suppressor mechanism, which promotes cancer cell proliferation and tumourigenesis, but on the other hand, autophagy favours cellular survival in low-oxygen/nutrient conditions or in response to toxic insults such as chemotherapeutic drugs or ionizing radiation (IR).^12, 13^

Various preclinical models revealed that autophagy is activated in irradiated tumour cells^14, 15, 16^ and that ATG expression patterns are upregulated following irradiation.^15^ However, the role of autophagy in the resistance of cancer cells to radiation therapy remains controversial. Preclinical studies indicated that the sensitivity of tumour cells to radiation therapy could be enhanced by co-treatment with autophagy inducers, such as rapamycin, both *in vitro* and in lung cancer xenograft models.^17^ Consistently, treatment with autophagy inhibitors, such as 3-methyladenine, is associated with reduced sensitivity to radiation in models of advanced papillary thyroid cancer.^18^ In sharp contrast, autophagy induction contributes to radiation resistance of CD133^+^ tumour cells, suggesting that autophagy inhibitors could be employed to radiosensitize cancer cells.^19^

In this context, we decided to (re-)evaluate the role of autophagy during tumour irradiation. Here, we report that irradiation-induced autophagy exerts a dual activity on tumour cell death and on tumour clearance by the immune system. On one hand, autophagy increases tumour cell survival by inhibiting cell death induction. However, on the other hand, autophagy contributes to the release of cell death-associated danger signals that trigger anti-tumour host immune responses.

## Results

### Autophagy inhibition reduces clonogenic survival after IR

**Figure 1:**
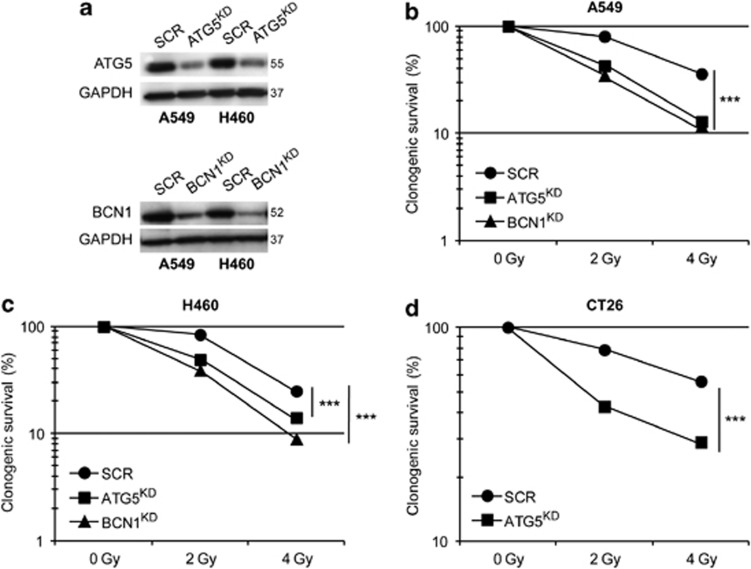
Knockdown of ATG5 or BECN 1 alters autophagy after irradiation and enhances radiation sensitivity of A549, H460 and CT26 cells. (**a**) Decreased ATG5 and BECN1 expression in A549 and H460 cells by RNA interference was observed by immunoblot. A representative immunoblot of three independent experiments is shown. (**b**–**d**) Cell lines as indicated. Radiation sensitivity of ATG5- and BECN1-depleted cells was assessed by clonogenic assay. Cells were transfected with a control shRNA or with an shRNA-targeting ATG5 or BECN1, then cells were left untreated or irradiated by 2 or 4 Gy. Cellular colonies were coloured and counted after 12–14 days and survival fraction (SF) was calculated and represented as described before. Quantitative data are reported as means±S.E.M. (*n*=3 triplicate samples, Student's *t*-test, ***P<0.001, as compared with untreated cells)

Autophagy has previously been associated with either cell survival or cell death promoting mechanisms. Thus, we decided to determine the role of autophagy during IR. To this aim, we analysed the effect of ATG5 or Beclin 1 depletion in H460 and A549 cells. We also analysed the effect of ATG5 depletion in CT26 cells. Using short hairpin RNA (shRNA) we silenced the expression of ATG5 in A549, H460 and CT26 cells, and observed that following IR with 2 or 4 Gy, the survival fraction (SF) determined by clonogenic assays was significantly reduced, as compared with cells that express unrelated shRNA controls (SCR) and thus had normal levels of autophagy. Thus, the inhibition of autophagy sensitizes A549, H460 and CT26 cells to IR-induced cell death (Figures 1b–d). Following the same experimental procedure, we found that silencing of Beclin 1, yet another autophagy-relevant gene, in A549 and H460 cells significantly decreased the clonogenic survival following IR (Figures 1b and c), corroborating that autophagy indeed operates as a cytoprotective mechanism that counteracts irradiation-induced cell death.

### ATG5 depletion induces cell death following IR

**Figure 2:**
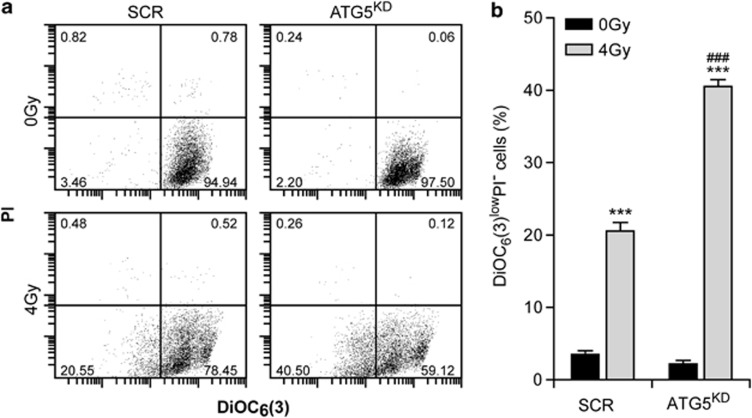
IR-induced cell death is increased in ATG5-depleted cells. (**a**) ATG5 depletion triggers mitochondrial membrane depolarization of A549 cells after ionizing radiation. A549 cells transfected by scrambled siRNA (SCR) or by specific shRNA for ATG5 (ATG5^KD^) were irradiated by 4 Gy and then subjected one day later to dual staining with the vital propidium iodide (PI) dye and with the mitochondrial transmembrane potential-sensitive dye 3,3 dihexyloxacarbocyanine iodide DiOC_6_(3) dye. Cell death was quantified by cytofluorometric analysis. (**b**) Histogram reporting the experiment shown in (**a**). Quantitative data are shown as means±S.E.M. (*n*=3 triplicate samples, Student's *t*-test, ***P<0.001 comparing ATG5^KD^ (4 Gy) with ATG5^KD^ (0 Gy) and SCR (4 Gy) with SCR (0 Gy); ^###^*P*<0.001 comparing ATG5^KD^ (4 Gy) with SCR (4 Gy))

Given that autophagy inhibition reduced clonogenic survival following IR, we wondered whether cell death induction would be required for the reduction of clonogenic survival. To evaluate the contribution of different cell death modalities to this process, we simultaneously monitored the dissipation of the mitochondrial membrane potential (Ψ_m_) and the loss of plasma membrane integrity, which are indicative for apoptotic or necrotic events. We observed that IR of A549 cells with 4 Gy induced a decrease in Ψ_m_ (as revealed by the loss of DiOC_6_(3) staining (DiOC_6_(3)^low^) in the absence of plasma membrane permeabilization (PI^negative^) indicating induction of lethal stress. The knockdown of ATG5, which abolishes autophagy (data not shown), led to a significant increase in the PI^-^DiOC_6_(3) ^low^ population of cells after 6 h of irradiation with 4 Gy (Figures 2a and b), indicating that the presence of ATG5 protects irradiated cells from death.

### Depletion of ATG5 or Beclin 1 radiosensitizes human cancers xenografted on immuodeficient mice

**Figure 3:**
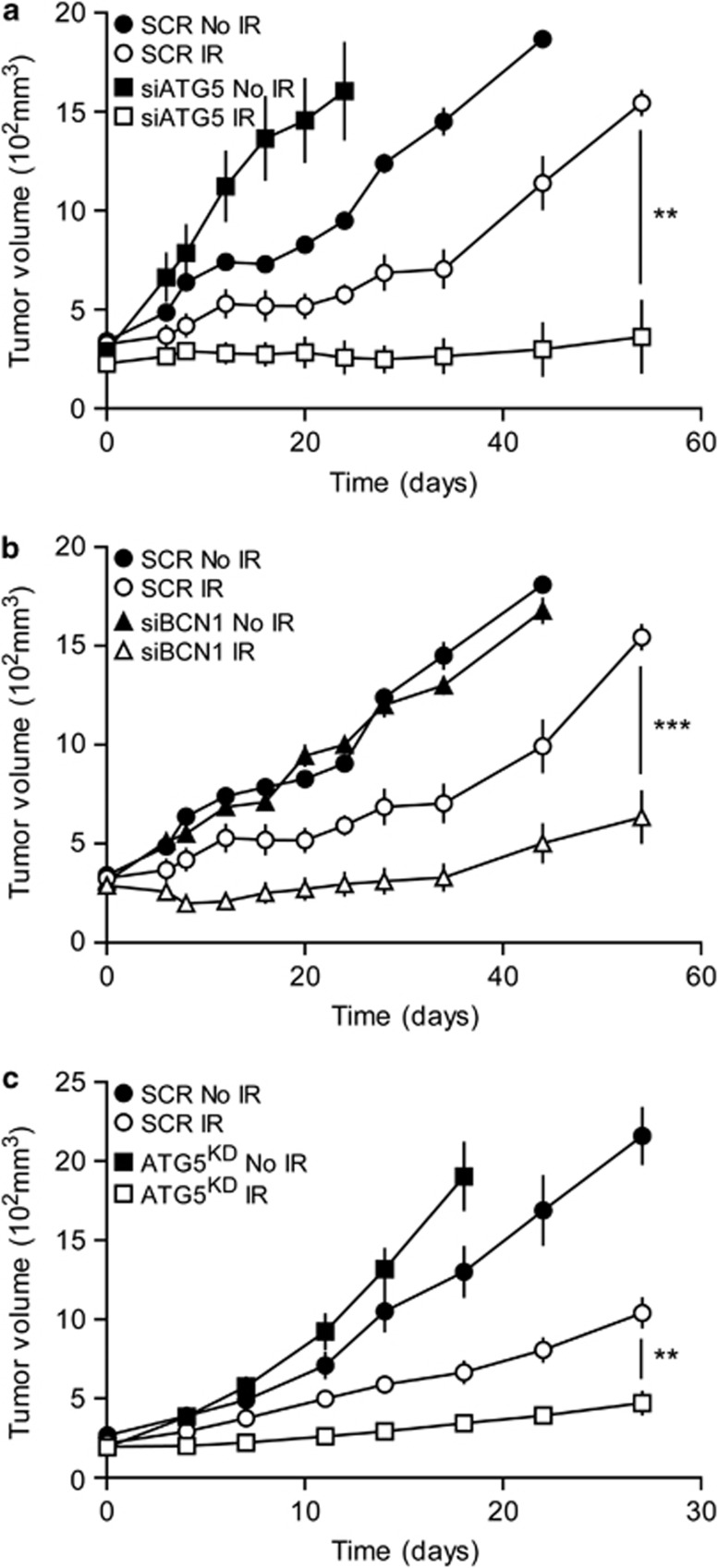
Autophagy delays radiation-induced growth *in vivo*. (**a**–**c**) Effects of ATG5 or BECN1 knockdowns on the growth of irradiated A549 or H460 tumours. Five nude BALB/c mice were subcutaneously injected with ATG5- or BECN1-depleted A549 cells (**a** and **b**) or H460 cells (**c**). Once xenografted tumours became palpable, mice were randomly assigned to a control group or to a group that received localized tumour irradiation with a single dose 8 Gy. Then, tumour volumes were monitored every 2–4 days. Quantitative data are reported as means±S.E.M. (Student's *t*-test, **P<0.01, ***P<0.001, as compared with the control group)

Considering that IR-induced tumour cell death *in vitro* fails to explain some clinical observations *in vivo*, we decided to evaluate the effect of ATG5 and Beclin 1 depletion on the growth of irradiated A549 tumour xenografts implanted in immunodeficient BALB/c nude mice (which lack T lymphocytes). As expected, control shRNA-transfected A549 tumours (SCR) presented a significant reduction in tumour size, as measured 24 days or 6 weeks after 8 Gy irradiation. This effect was significantly enhanced when autophagy-relevant genes ATG5 (Figure 3a) or Beclin 1 (Figure 3b) were knocked down. Then, we calculated the absolute growth delay (AGD) and the dose-enhancement factor (DEF) after irradiation to determine the impact of autophagy impairment on tumour growth after irradiation. AGD can be defined as the difference of time (determined in days) for non-irradiated *versus* irradiated tumours to reach a 50% volume increase. The DEF score was calculated as the difference in normalized tumour growth delay between irradiated autophagy-deficient *versus* irradiated autophagy-proficient tumours. We found that AGD was 4, 49 and 39 days for control (SCR), ATG5-deficient (ATG5^KD^) and BECN-1-deficient (BECN-1^KD^) tumours, respectively. DEF was 4.5 for ATG5^KD^ tumours and 3.7 for BECN-1^KD^ tumours, revealing ATG5 or BECN-1 depletion sensitized A549 tumours to IR.

Next, we evaluated the impact of irradiation-induced autophagy on the growth of xenografted H460 tumours. Similar to our previous results, control transfected H460 tumours (SCR) presented an average tumour volume of 293±29 and 588±49 mm^3^ after 5 and 15 days of irradiation, respectively. In contrast, non-irradiated tumours revealed a mean tumour volume of 391±42 and 1050±124 mm^3^ after 5 and 15 days of growth, respectively. ATG5-defective H460 (ATG5^KD^) tumours were sensitized to irradiation and detected a significant enhancement in irradiation-induced tumour growth delay compared with autophagy-competent control tumours (Figure 3c). As compared with non-irradiated xenografts, the mean tumour volume of ATG5^KD^ tumours was significantly reduced 15 days after irradiation. Altogether, these results revealed that autophagy favours the growth of irradiated tumours in immunodeficient mice.

### ATG5 depletion impairs efficiency of tumour growth immunomodulation

**Figure 4:**
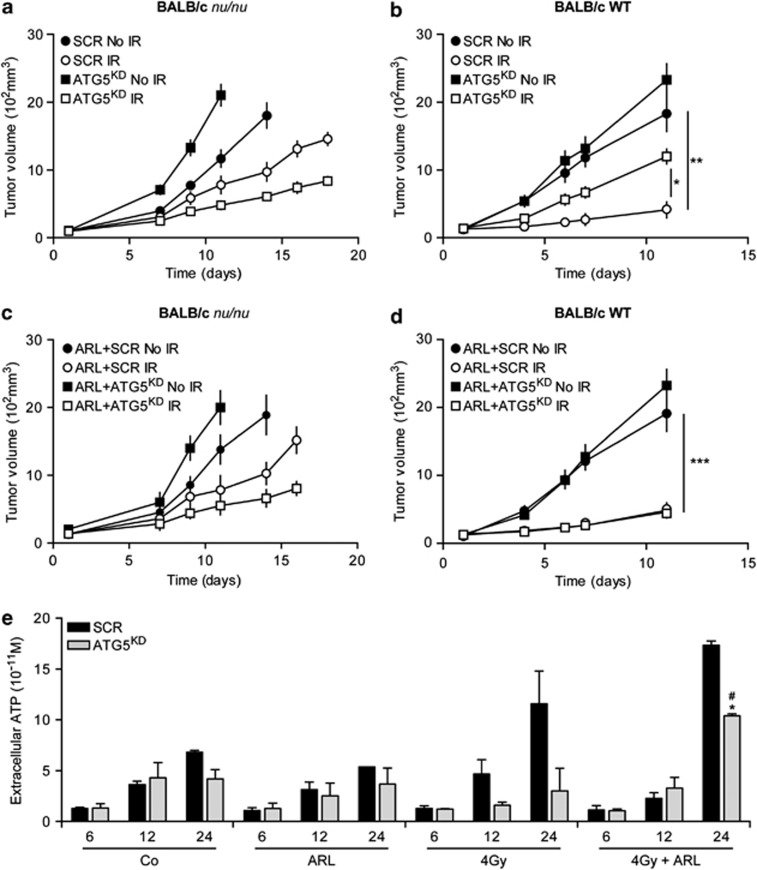
Autophagy inhibition decreases irradiation-induced ATP release and impairs anti-tumoural immune response. (**a**–**c**) Role of ATG5 on the growth of irradiated murine CT26 tumours implanted on immunodeficient mice. Nude BALB/c mice were subcutaneously injected with SCR or ATG5-depleted CT26 cells, then irradiated or left untreated with (**c**) or without an inhibitor of ecto-ATPase ARL67156 (ARL) (**a**). Tumour volumes were monitored every 3 or 4 days by direct measurement, as previously described. (**b**) Same experiment as in (**a**) in an immunocompetent host. Wild-type BALB/c mice were subcutaneously injected with ATG5-depleted CT26 cells, left untreated or irradiated, then tumour volume was monitored. Quantitative data presented are reported as means±S.E.M. (Student's *t*-test, *P<0.05, **P<0.01). (**d** and **e**) Tumour growth of irradiated tumours is inhibited by ATP release via autophagy. To determine the impact of irradiation and autophagy on ATP release, ATG5depleted CT26 (ATG5^KD^) or control (SCR) cells were irradiated with 4 Gy and incubated with or without an inhibitor of ecto-ATPase ARL67156 (ARL). ATP release was determined *in vitro* by using enzymatic methods at indicated times (**e**). Quantitative data are reported as means±S.E.M. (*n*=3 triplicate samples, Student's *t*-test, *P<0.05 comparing ATG5^KD^ (4 Gy+ARL) with ATG5^KD^ (Co) at 24 h; ^#^*P*<0.05 comparing ATG5^KD^ (4 Gy+ARL) with ATG5^KD^ (4 Gy) at 24 h). To assess the impact of extracellular ATP release by irradiation on tumour growth, Balb/c mice were injected with scramble or Atg5-depleted CT26 tumour cells, then xenografted tumours were randomly assigned to treatment groups that received localized tumour irradiation with one single 8 Gy irradiation, with ARL (**d**). Tumour volumes were determined as indicated above and quantitative data are reported as means±S.E.M. (Student's *t*-test, ***P<0.001)

Numerous preclinical studies suggest that the activation of the immune system is essential for successful radiotherapy.^20, 21, 22^ To evaluate the role of IR-induced autophagy on anti-cancer immune responses, we analysed tumour growth of autophagy-deficient (ATG5^KD^) or -proficient (SCR) colon carcinoma CT26 cells (which are derived from BALB/c mice) in immunodeficient and immunocompetent BALB/c mice. Similar to our previous results with human tumour xenografts, knockdown of autophagy genes significantly enhanced the radiation-induced tumour growth delay for CT26-derived tumours growing on immunodeficient mice. Thus, in the context of an absent cellular immune response, ATG5-deficient (ATG5^KD^) tumours were significantly more sensitive to IR than autophagy-competent control tumours, as revealed by the comparison of tumour volumes measured before and after 10 days of irradiation (Figure 4a). In sharp contrast, autophagy inhibition resulted in a reduced tumour growth-inhibitory effect of radiotherapy, if autophagy-deficient or -competent cancers were implanted into immunocompetent BALB/c mice and then irradiated (Figure 4c). Taken together, these findings suggest that IR-induced autophagy has a major role in the onset of anti-cancer immune responses.

Following irradiation, the induction of immunogenic cell death (ICD) can lead to the emission of danger signals that are exposed at the cell surface or are released into the extracellular space, thus stimulating anti-tumour immune responses.^23, 24^ Adenosine triphosphate (ATP) is one of the danger signals released during ICD.^25, 26^ Considering that the onset of autophagy is associated with ATP release after chemotherapy,^27^ we decided to evaluate the effect of IR-induced autophagy on ATP release. Twenty-four hours post irradiation, autophagy-deficient (ATG5^KD^) mouse colon carcinoma CT26 cells released significantly less ATP than their autophagy-competent counterparts (Figure 4e), demonstrating that IR-induced autophagy indeed modulates IR-induced ATP release. In line with previously published observations,^27^ administration of the ecto-ATPase inhibitor ARL67156 to CT26 cells increased the extracellular concentration of ATP after 4 Gy irradiation *in vitro* (Figure 4e). In addition, intratumoural injection of ARL67156 increased the radiosensitivity of ATG5-deficient (ATG5^KD^) tumour cells in an immunocompetent context, indicating that it restored the immune response to irradiated ATG5^KD^ CT26 tumour cells (Figure 4d). The tumour growth-inhibitory effect of ARL67156 was lost in immunodeficient mice, indicating that it entirely relies on the presence of an intact immune system (Figure 4b).

**Figure 5:**
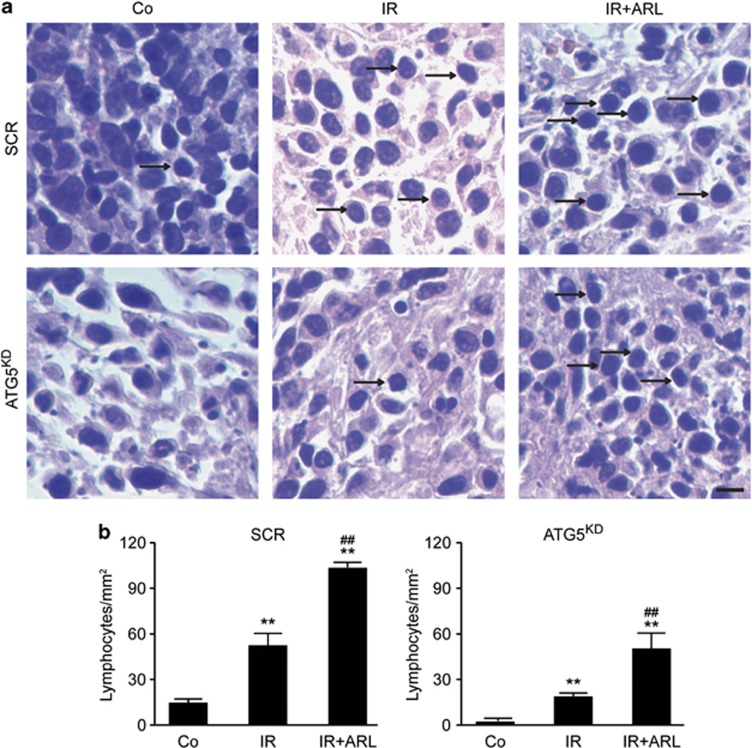
ARL67156 restores the lymphocyte infiltration in Atg5-depleted CT26 tumour after irradiation. (**a**) Representative pictures of CT26-recovered tumours. Scale bar, 10 *μ*m. Arrows point lymphocytes. Quantitative data are reported in (**b**). Samples were compared using one-tailed Student's *t*-test. Error bars indicate S.E.M. **P<0.01, comparing SCR (IR) with SCR (Co) and SCR (IR+ARL) with SCR (Co); ^##^*P*<0.01, comparing SCR (IR+ARL) with SCR (IR); **P<0.01 comparing ATG5^KD^ (IR) with ATG5^KD^ (Co) and ATG5^KD^ (IR+ARL) with ATG5^KD^ (Co); ^##^*P*<0.01 comparing ATG5^KD^ (IR+ARL) with ATG5^KD^ (IR) in (**b**)

Nine days post irradiation, autophagy-competent tumours were more heavily infiltrated by lymphocytes than autophagy-deficient cancers, as revealed by histological analysis. Intratumoural injection of ARL67156 into ATG5-deficient (ATG5^KD^) tumour cells significantly enhanced lymphocyte recruitment into irradiated ATG5^KD^ CT26 cancers (Figure 5), commensurate with the improved growth-inhibitory action of irradiation on such tumours.

## Discussion

For decades, apoptosis has been considered as the principal cell death pathway elicited by radiotherapy, although numerous preclinical studies questioned this predominance.^28^ *In vivo* experiments have shown that mice lacking essential pro-apoptotic effectors (such as Bax and Bak, caspases, Apaf-1 or caspase-9) exhibit relatively conserved phenotypes and do not completely suppress homeostatic programmed cell death.^29^ Non-apoptotic cell death morphologies or alternative death mechanisms have been discovered during radiation treatments, including ‘autophagic cell death', programmed necrosis, mitotic catastrophe and entosis (also called cellular cannibalism).^30, 31^ Recently, some preclinical results have highlighted the contribution of autophagy to radiation-induced cell death.^15, 32^ Autophagy is a lysosomal degradation mechanism that is known to be essential to cellular homeostasis, as it is involved in degradation of misfolded proteins and removing dysfunctional organelles such as mitochondria.^33^ Thus, autophagy acts as a stress-responsive pathway that may confer protection against external stress to promote cell survival.^34^ Indeed, there is accumulating evidence that autophagy is upregulated in response to chemotherapy, radiation therapy or hypoxia.^35^

The role of autophagy in radiation-induced cell death is barely understood. Some researchers suggested that simultaneous activation of apoptosis and autophagy enhances the sensitivity of radiation therapy both *in vitro* and in lung cancer xenograft models.^17^ In contrast, short-time inhibition of autophagy was reported to increase the cytotoxicity of radiotherapy in resistant cancer cells.^15^ On the basis of these results, we wondered whether combining autophagy inhibition with radiation therapy would enhance the efficiency of anti-tumour treatment. Clonogenic assays showed that knockdown of two different ATG (*BECN1* and *ATG5*) had a radiosensitizing effect, suggesting that autophagy can prevent radiation-induced cell death. Consistent with data from other researchers,^36, 37, 38^ we found that autophagy inhibition enhanced tumour growth *in vivo*. However, as compared with autophagy-competent cancers, tumour cells with defective autophagy were significantly more sensitive to irradiation, *in vivo*, in an immunodeficient context.

The impact of inflammatory and immune responses on tumour progression is controversial. On one hand, chemokines secreted by tumours can promote tumour progression indirectly by stimulating leukocytes to secrete survival and neo-angiogenesis signals.^39^ On the other hand, dendritic cells have a major role in stimulating the anti-cancer immune response as they process tumour antigens and present them to other cells of the immune system.^40^ In specific circumstances, cancer cells can undergo immunogenic apoptosis, meaning that their corpses are engulfed by dendritic cells and their antigens are subsequently presented to tumour-specific CD8^+^ T cells.^41^ Some successful chemotherapeutics induce immunogenic cell death, implying that the patient's dying cancer cells are converted into a therapeutic vaccine. Immunogenic cell death is characterized by the pre-apoptotic exposure of calreticulin (CRT) on the cell surface, post-apoptotic exodus of the chromatin-binding protein high-mobility group B1 (HMGB1) and release of ATP. Autophagy is not required for chemotherapy-induced CRT exposure and HMGB1 release,^27^ and our preliminary data suggest that autophagy is not required for radiation-induced CRT exposure or HMGB1 release either. Autophagy is strictly required for the release of ATP from dying tumour cells,^27^ which in turn is required for attracting immune cells including DC into the tumour.^42^ These results have been obtained in the context of immunogenic anti-neoplastic therapies.^27, 42^ Here, we report that, as compared with their autophagy-competent counterparts, autophagy-deficient cancer cells released reduced amounts of ATP also in the context of radiotherapy.

In this study, we wondered whether autophagy-dependent immunogenic signalling might affect the efficacy of radiotherapy. In experiments involving tumours implanted in immunodeficient nude mice, the radiosensitivity of ATG5^KD^-deficient cancers was higher than that of autophagy-competent controls. In stark contrast, however, autophagy-deficient tumours were less radiosensitive than control tumours in an immunocompetent context if the tumours evolved on BALB/c wild-type mice. The failure of IR-treated cancer cells depleted from Atg5 to induce a protective immune response *in vivo* could be corrected by intratumoural injections of ARL67156 (ARL), an inhibitor of ecto-ATPases (apyrases) that artificially increases extracellular ATP concentrations.^43^ ARL injections enhanced the efficacy of IR therapy against autophagy-deficient CT26 cancers, hence significantly reducing tumour growth. As a control, ARL had no effect on autophagy-competent CT26 cancers. We conclude from these experiments that radiation-induced immunogenic cell death is associated with the autophagy-dependent extracellular accumulation of ATP.

In summary, this work underscores the important role of autophagy in the radiation response, both at the cell autonomous and at the immunological levels. To date, it is impossible to establish a link between autophagy and radiosensitivity without taking into account the immunological tumour microenvironment.

## Materials and Methods

### Cell lines

Human non-small-cell lung cancer cell lines A549 and H460, as well as mouse colon adenocarcinoma CT26 cells, were obtained from ATCC (Manassas, VA, USA). A549 cells were cultured in DMEM-F12 (Life Technologies, Carlsbad, CA, USA) medium containing 10% fetal bovine serum (FBS), 1% penicillin/streptomycin (PS) and 0.1% puromycin. H460 cancer cells were maintained in RPMI-1640 (Life Technologies) medium containing 10% FBS and 1% PS. CT26 cells were cultured in RPMI-1640 medium (Life Technologies) containing 10% FBS, 1% PS and 0.1% puromycin. All cells were maintained under 5% CO_2_ humidified atmosphere at 37 °C.

### RNA interference

To generate A549 and H460 cells that stably express short hairpin RNA (shRNA) targeting *ATG5* and *BECN1*, the corresponding constructs (obtained from Origene, Rockville, MD, USA) were transfected together with scrambled control vectors into retrovirus producing Phoenix ampho cells by means of Lipofectamin 2000 (Life Technologies) following the manufacturer's instructions. ShRNA-encoding viruses were used to infect A549 cells in the presence of 4 *μ*g/ml polybrene (Millipore, Billerica, MA, USA). Cells transduced with shRNA were selected in 3 *μ*g/ml puromycin (InvivoGen, San Diego, CA, USA) and single clones were obtained by limiting dilution. Insertion was validated by immunoblotting with the suitable antibodies. For the generation of autophagy-deficient CT26, a set of plasmids encoding shRNAs specific for murine Atg5 plus a control shRNA were obtained from Origen and used as described above.

Small interfering RNA control (UNR, universal negative control #1) or specific for human ATG5 (sense 5′-CCUUUGGCCUAAGAAGAAATTdTdT-3′) or for human BECN1 (sense 5′-CAGUGGAUCUAAAUCUCAATTdTdT-3′) were purchased from Sigma-Proligo (The Woodlands, TX, USA). H460 and A549 cells were transfected (at 30–40% confluence) with the HiPerFect transfection reagent (Qiagen, Hilden, Germany)—previously complexed with 100 nM siRNA—as instructed by the manufacturer. Transfected cells were used for experiments no earlier than 48 h after transfection. Protein knockdown was confirmed by immunoblotting.

### Western blot analysis

A549 or H460 cells were washed with PBS, then exposed to cell lysis buffer containing 100 mM NaCl, 50 mM Tris-HCl pH 7.4, 5 mM EDTA pH8, 50 mM NaF, 1% IGEPAL CA-630 (Sigma-Aldrich, St. Louis, MO, USA), 1 mM NaOV (phosphatase inhibitor), 1X protease inhibitor (Boehringer, Ingelheim-Am-Rhein, Germany). After protein extraction on ice, protein dosage was performed using the Bio-Rad Protein Assay Kit (Bio-Rad, Hercules, MA, USA) as recommended by the manufacturer, then the protein concentration was measured using the Bio-Rad microplate reader Model 680 system equipped with Microplate manager 5.2 software (Bio-Rad). Following denaturing at 95 °C for 5 min, 30 *μ*g of proteins were loaded onto denaturing acrylamide gel at 4–12% (NuPaAGE Bis-tris Gel, Life Technologies). Migration was performed at 200 V for 90 min. Proteins were blotted onto nitrocellulose by electro-transfer at 40 V for 1 h. Membranes were washed with TBS containing at 0.1% Tween-20 (TBS-T) and then blocked for 1 h in TBS-T containing 5% bovine serum albumin. The membranes were incubated overnight at 4 °C with the following primary antibodies: anti-ATG 5 (from Cell Signaling Technologies, Danvers, MA, USA) and anti-BECN1 (from Santa Cruz, CA, USA). Horseradish peroxidase-labelled goat anti-rabbit or anti-mouse antibodies from GE Healthcare (Buckinghamshire, UK) were used for signal detection with direct scanning (Image Quant 350, GE Healthcare). Measurements were normalized by assessing expression of glyceraldehyde-3-phosphate dehydrogenase (GAPDH, from Biodesign International, Saco, ME, USA).

### Clonogenic survival and combination studies

Transfected and untransfected cells were plated at different cellular density. The day after, cells were irradiated at doses ranging from 0 to 4 Gy (Cesium=Cs^137^, 1 Gy/min, gamma irradiator IBL-637 from CIS-BioInternational, IBA, Saclay, France). Then, cells were incubated for 10–12 days under standard culture conditions and thereafter, cellular colonies were coloured with crystal violet, washed and counted. Only cellular colonies with more than 50 cells were taken into consideration. Each surviving fraction (SF) was calculated by dividing the number of cellular clones by the number of cells plated and was further normalized based on the ratio of clonogenic survival for each radiation dose and clonogenic survival of non-irradiated controls. The sensitivity enhancement ratio for each radiation dose was calculated by dividing the SF of transfected cells by the SF of untransfected cells.

### Cell death detection

Scrambled and *ATG5* shRNA expressing cells were irradiated 24 h after plating. After 6 h, cell death was quantified by cytofluorometric analysis using a FACS Vantage (Becton Dickinson, Mountain View, CA, USA). Thus, cells were stained with 40 nM 3,3 dihexyloxacarbocyanine iodide (DiOC_6_(3); Molecular Probes, Eugene, OR, USA) for 30 min at 37 °C and concomitantly with 1 *μ*g/ml propidium iodide (PI; Sigma-Aldrich) for 30 min at 37 °C to determine the mitochondrial transmembrane potential. Data were statistically evaluated using CellQuest Pro software (BD Biosciences, San Diego, CA, USA).

### *In vivo* assessments

A549, H460 and CT26 cells stably transfected with a shRNA-targeting ATG5 or BECN1 were grown as tumour xenografts in the right flank of nude or BALB/c mice aged from 6 to 8 weeks from Janvier CERT (Le Genest, St. Isle, France). Tumour-bearing mice were randomly assigned to treatment groups (at least six in each group) and mice from the irradiation group received localized tumour irradiation with single dose IR (8 Gy) using 225 kV COMET MRX-225/22 X-ray (Stamford, CT, USA) at a dose rate of 0.35 Gy/min (200 kV, 15 mA, 0.2 mm Cu filter). Control groups received no irradiation. Tumour size and body weight were measured twice a week. Tumour volume was calculated using the formula: (length × width^2^)/2. Mice were sacrificed when the tumour volume reached 1500–2000 mm^3^ (Figures 3 and 4) or at day 9 after IR (Figure 5).

### Animal experimentations

Animals were maintained in specific pathogen-free conditions, and experiments followed the Federation of European Laboratory Animal Science Association (FELASA) guidelines. Animal experiments were approved by the local Ethics Committee (CEEA IRCIV / IGR n°26, registered with the French Ministry of Research) and were in compliance with EU 63/2010 directive. Animals were used between 6 and 20 weeks of age and those bearing tumours exceeding 20–25% body mass were killed. BALB/c (H-2d) mice were obtained from Janvier (Le Genest). The *in vivo* experiments were carried out under the animal care licence no. D94-076-11 (Ministère de l'Agriculture).

### ATP release assays *in vitro*

Extracellular ATP levels were measured by the luciferin-based ENLITEN ATP Assay (Promega, Fitchburg, WI, USA) and ATP assay (Calbiochem, Nottingham, UK) kits, respectively, in excess of luciferin and luciferase, as indicated by the manufacturer.

### Histology and HES staining

Recovered tumours were fixed with 4% PFA for 3 h and then embedded into paraffin. Sections of 4 *μ*m paraffin were fixed and stained with haematoxylin and eosin according to standard protocols.

## Abbreviations

AGD: absolute growth delay
ATG: autophagy-related gene
ATP: adenosine triphosphate
BECN-1: beclin-1
CRT: calreticulin
DC: dendritic cell
GAPDH: glyceraldehyde 3-phosphate dehydrogenase
HMGB1: high-mobility group 1
IR: ionizing radiation
LC3: microtubule-associated protein 1A/1B light chain 3
MAP: mitogen-activated protein
PE: phosphatidylethanolamine
PI3K: phosphatidylinositol 3-phosphate kinase
SCR: scramble
SF: surviving fraction
shRNA: short hairpin interfering RNA
siRNA: small interfering RNA
WT: wild type
